# Evaluation of Aneurysm Cases Undergoing Surgery at a Tertiary Center in Iran: A 22‐year Retrospective Study

**DOI:** 10.1002/hsr2.70331

**Published:** 2025-01-07

**Authors:** Hamed Ghoddusi Johari, Keivan Ranjbar, Kimia Kassaee, Seyed Mohammadali Hoseini, Reza Shahriarirad

**Affiliations:** ^1^ Vascular Surgery Department Shiraz University of Medical Sciences Shiraz Iran; ^2^ Thoracic and Vascular Surgery Research Center Shiraz University of Medical Sciences Shiraz Iran; ^3^ Division of Vascular Surgery, Cardiovascular Center Tufts Medical Center Boston Massachusetts USA; ^4^ School of Medicine Iran University of Medical Sciences Tehran Iran; ^5^ Student Research Committee Shiraz University of Medical Sciences Shiraz Iran

**Keywords:** aneurysm, rupture, surgery, vascular surgery

## Abstract

**Background and Aims:**

An arterial aneurysm is characterized by a localized expansion of a blood vessel relative to its original dimensions. Specifically, an abdominal aortic aneurysm (AAA) is identified as an aortic diameter measuring at least one and a half times the standard diameter at the renal artery level, approximately equivalent to 2.0 cm. In this study, we aim to evaluate the prevalence of AAA, along with the clinical features, trend, and incidence of ruptured AAA among patients undergoing surgery in our center.

**Methods:**

The database of patients operated in Namazi Hospital from 2000 to 2021 was retrieved and patients undergoing vascular surgeries due to aneurysm were reviewed. All data were analyzed with SPSS version 26.0.

**Results:**

A total of 599 cases of aneurysm were operated, among which 334 were contributed to the aorta and included in our study. The average age of the participants was 69.6 (SD: 12.1, range 16–93) years and 161 (85.2%) were male. The majority of cases were in the 60 to 80 years age group (*n* = 205; 62.5%). There was a significant association between the age groups and the AAA rupture (*p* = 0.003), with the highest occurrence among the above 80 years age group (*n* = 37, 49.3%). Regarding the location of the aneurysm, 274 were located in the infrarenal and abdominal region, 21 in the thoracoabdominal region, and 12 in the thoracic region. Among the cases in our study, 112 were cases of ruptured aneurysms. Furthermore, the age of patients with ruptured aneurysm were significantly higher compared to non‐ruptured patients (71.8 vs. 68.5; *p* = 0.019).

**Conclusion:**

We observed an increase in the incidence of AAA surgeries in our center throughout the years, with the population growing towards younger population, while the incidence of rupture increasing towards older age groups.

## 1 Introduction

Abdominal aortic aneurysm (AAA) is a serious vascular pathology characterized by the progressive dilation of the abdominal aorta, often remaining asymptomatic until rupture that can lead to life‐threatening complications. Ruptured AAAs carry a mortality rate as high as 65%–80% [1, 2, 3]. AAA occurs when there is a loss of elastic tissue and smooth muscle cells in the aorta, which may be caused by inflammation and the presence of certain enzymes [4, 5].

AAA is a condition which affects a great proportion of the population, in which current smokers face a higher risk [6]. Smoking is known to be the most important risk factor for AAA, which is associated directly with development, growth and rupture of AAAs. It elevates AAA growth rate by 15%–24% and escalates the risk of rupture, regardless of diameter [7, 8, 9, 10, 11]. Age is a significant risk factor in AAA development, with the risk increasing nearly 200‐fold for a 75–79‐year‐old man compared to a 40‐44‐year‐old man [12]. Although data regarding AAA incidence in women is less extensive, studies have shown that the risk of developing an AAA in women increases with age in a similar way, but at a lower level [1, 13]. Women are likely to develop AAAs later than men, but they experience a faster progression and a four times higher risk of rupture than men [11, 14, 15].

A study from 1991 to 2013 found prevalence rate of 6.0% for men as opposed to 1.6% for women, although reported incidence and prevalence rates vary due to AAA definition and study populations [1, 16]. Even when treated, patients with AAA have a higher mortality rate than the general population [17]. Although rupture is the main complication of AAAs, cardiovascular events are the most common causes of death among AAA patients [18, 19]. Numbers show that AAA incidence, prevalence, and mortality has decreased in the past two decades, especially in North America and Western Europe. This trend is largely attributed to smoking cessation efforts and the introduction of screening programs for high‐risk populations [12, 20].

The decision whether to perform surgery on AAA is a tricky one and is indicated when rupture risk is higher than surgical risk, in which approximately 85% of AAA repairs are performed for intact aneurysms electively. Current guidelines recommend elective repair for asymptomatic AAAs with a diameter of 5.5 cm in men and 5.0 cm in women, if the surgical risk is not high [21, 22]. When the aneurysm ruptures an immediate repair is performed with the significant mortality rate up to 85% [23, 24, 25].

The options for repair consist of open surgical repair, aiming to replace the aneurysmal wall by a synthetic graft, and endovascular repair (EVAR), which involves the insertion of an endograft into the lumen to exclude the aneurysm from the systemic circulation [26, 27]. The choice of whether to perform open surgical repair or EVAR depends mainly on patient characteristics and the patient's willingness to attend follow‐up sessions [28, 29, 30, 31]. However, both approaches are similar regarding their long‐term outcomes [32].

This study aims to investigate the epidemiological and clinical features of surgical aneurysm cases managed by the vascular surgery service in a referral center in southern Iran from 2000 until 2021. Identifying these characteristics can provide a better understanding of patient risk factors, procedural outcomes, and treatment approaches, forming a basis for further research into the optimal management of aneurysms.

## 2 Materials and Methods

In this retrospective, hospital record‐based study, all operative notes of patients undergoing surgery at Namazi Hospital, affiliated with Shiraz University of Medical Sciences during a 22‐year period (May 2000 to August 2021) were evaluated. Shiraz is the largest city in southern Iran and the Namazi Hospital is the largest referral medical center in the south of Iran and neighboring countries, with 32 general and specialized wards. The medical center comprises 600 beds, and its bed occupancy rate stands at 85%. The Namazi Operation room the main center which provides vascular and endovascular surgeries in Southern Iran.

Among the recorded data, vascular surgeries were selected and separated based on the attending physician specialty. The operative features of these patients were evaluated along with their diagnosis, and those unrelated to vascular surgery were excluded. Cases with a diagnosis of aneurysm were included in our study. Keywords used for data extraction included: “Aneurysm” OR “Aortic” OR “AAA” OR “Aorto‐ “OR “Aorta” AND “Ruptured.”

Diagnosis of AAAs was achieved through a standardized protocol involving clinical assessment and imaging [21]. Initial evaluation included a thorough patient history focusing on risk factors along with a physical examination for abdominal pulsations. Ultrasound was the first‐line imaging modality, particularly for elder men who had history of smoking. If further detail was necessary, CT angiography was used for surgical planning. Also, follow‐up imaging intervals were based on aneurysm size.

Obtained data included patients age, gender, admission and post‐surgery ward, type of operation, attending surgeon, preoperative and post‐operative diagnosis, and operation findings. These data were recorded in an excel worksheet and evaluated. There was no age limitation in our study.

### 2.1 Data Analysis

Data were subsequently transferred to SPSS version 26.0 and analyzed accordingly. The normality of the data was checked with the Shapiro‐Wilk test. Quantitative data are presented as mean and standard deviation (SD) and qualitative data will be presented as frequency and percentage (%). A *P*‐value of less than 0.05 was considered significant.

## 3 Results

During the 22‐year period of our study, a total of 226,051 operations were performed at Namazi Hospital Operating room. Among these operations, 6859 (3.03%) operations were operated by vascular surgeons. In further evaluation of these operations, 473 were excluded due to being unrelated to vascular surgery and a final count of 6386 (2.82%) operations were evaluated.

**Figure 1:**
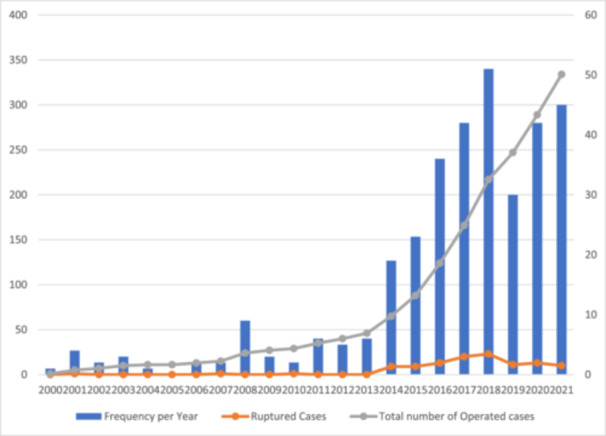
Frequency of operated cases of aneurysm throughout the years.

Based on our evaluation, a total of 599 cases of aneurysm were operated, among which 334 were contributed to the aorta and included in our study. Figure 1 demonstrates the time trend of operated cases throughout the period of our study. The average age of the participants was 69.6 (SD: 12.1, range 16–93) years and 161 (85.2%) were male, while 28 (14.8%) were female.

**Figure 2:**
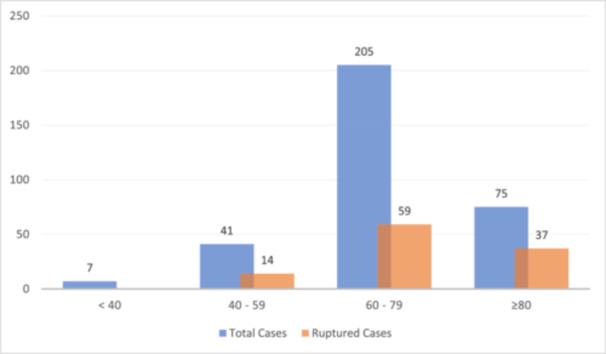
Frequency of total and ruptured abdominal aortic aneurysm based on age groups.

Based on the evaluation of the patients age groups, the majority of cases were in the 60 to 80 years age group (*n* = 205; 62.5%), followed by the 80 and above age group (*n* = 75; 22.9%), the 40–59 age group (*n* = 41; 12.5%), and lastly the below 40 age group (*n* = 7; 2.1%). Figure 2 demonstrates the occurrence of AAA among various age groups. There was a significant association between the age groups and AAA rupture (*p* = 0.003), with the highest occurrence among the above 80 years age group (*n* = 37, 49.3% of patients in the age group).

**Figure 3:**
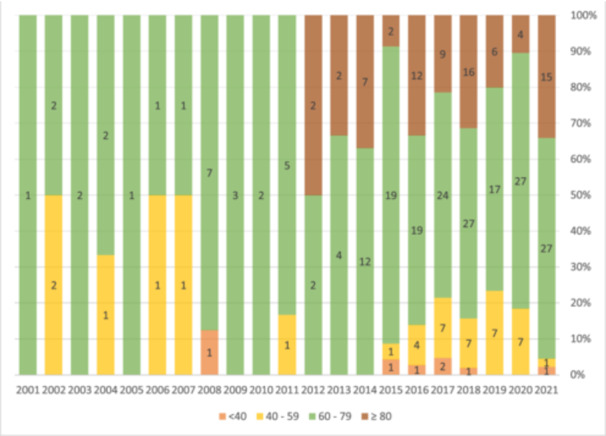
Evaluation of the trend of acute abdominal aneurysm based on age groups throughout the years.

We then evaluated the trend of AAA throughout the years based on age groups (Figure 3). As demonstrated, there seems to be an increasing trend toward older populations.

Regarding the location of the aneurysm, 274 were located in the infrarenal and abdominal region, 21 in the thoracoabdominal region, and 12 in the thoracic region.

In our study, 112 (33.5%) were cases of ruptured aneurysms. Based on the location, 86 of the cases of ruptured aneurysm were at the infrarenal and abdominal region, 12 at the thoracoabdominal, three at the thoracic. The average age of the patients was 71.9 (SD: 12.1; 40–93) years. Furthermore, the age of patients with ruptured aneurysm were significantly higher to non‐ruptured patients (71.8 vs. 68.5; *p* = 0.02). There was no significant association between ruptured aneurysms and gender (*p* = 0.45).

## 4 Discussion

We examined the trends in AAA from 2000 to 2021. During this timeframe, we computed rates and incidence of AAA, as well as occurrences of rupture. By using complete data from our operation room database, we successfully documented all surgical cases within a 22‐year span, covering a substantial population that includes the majority of AAA cases in the southern‐western region of Iran. Our assessment revealed several significant findings.

Prior epidemiologic studies based on data through 2000 have suggested an increasing or at least stable rate of AAA incidence, but these studies are now over 10 years old and outdated [33, 34, 35, 36, 37]. The population most likely to show decreased incidence of AAA are the youngest age groups who might have benefitted most from lower smoking rates, and other risk factor control [38]. Among the younger cohort (aged 65–74), we noted a reduction in the rupture rate, notwithstanding a decrease in intact repair rates. This decline in the rupture rate within this age group, coupled with a diminishing rate of intact repair, suggests a potential decline in the incidence of AAA in younger patients. In contrast, individuals aged 80 and older exhibited a substantial increase in intact repair rates along with a concurrent decline in ruptures. This notable surge in intact repairs in the older population implies that the prevalence of AAA is unlikely to be decreasing in this age group.

One of our key findings is the increasing trend of AAA among older patients. This aligns with other studies which reported that rates of intact AAA repair increased dramatically in those over age 80 [3, 39]. When evaluating results from other Iranian centers, a similar increase in the number of AAA repairs over a 10‐year period and a shift towards treating older patients was also noted [40]. In our center, we've seen a similar shift towards treating older patients, which presents challenges and opportunities for improving care.

The gender distribution in our study (85.2% male) is similar to that reported by other Iranian vascular centers. For example, a study from Tehran, affiliated with Shahid Beheshti University of Medical Sciences, reported that 84% of their AAA patients were male [40]. Men generally have a larger aortic diameter and may experience more significant atherosclerosis, increasing the risk of aneurysm formation.

Another finding from our study is that the incidence of AAA rupture has decreased over time, which is also in line with a study by Schermerhorn et al. [3]. As ruptures predominantly occur within the elderly demographic, this observation is likely attributed to the rising prevalence of prophylactic elective repairs in this particular age group. Furthermore, limited access to early screening and diagnostic tools, delayed presentation of patients, and differences in healthcare system might contribute to the higher rupture rates. Additionally, differences in lifestyle and healthcare‐seeking behaviors in Iran may increase the likelihood of aneurysms progressing undetected to rupture stages [41].

Kakisis et al. stated that ruptured AAA carries a 40%–80% mortality rate and over 1/3 of patients die before they reach a hospital [42]. In instances where the patient successfully reaches the hospital, employing an algorithmic strategy for stabilization, prompt diagnosis, and subsequent repair has demonstrated enhanced outcomes. Pre‐existing protocols for transfer to a center with experience in managing ruptures are also associated with improved outcomes [21]. The typical presentation involves the patient experiencing acute abdominal pain radiating to the back, accompanied by altered mental status due to hypovolemic shock. In cases where a high suspicion is warranted based on the patient's history and physical examination, immediate transfer to the operating room is recommended. If the diagnosis is uncertain and the patient's clinical condition allows, CTA or bedside duplex ultrasonography can be employed to establish a definitive diagnosis. Permissive hypotension is strongly recommended to minimize bleeding and coagulopathy [21, 42].

In 2015‐2016, we saw an increase in cases due to the addition of a new vascular surgeon to our team. This expansion of our expertize allowed us to treat more patients and likely improved access to care for many in our region. The COVID‐19 pandemic in 2020–2021 caused challenges, leading to a temporary drop in our statistics due to hospital closures and operation cancellations. However, we quickly adapted, prioritizing major operations and seeing a subsequent increase in AAA surgeries, which may cause a difference in rates with prior reports.

The study's limitations include the lack of detailed information on the patients, such as further demographical and clinical information. Also, the lack of follow‐up and outcome of the patients. Further longitudinal and cohort studies are required to obtain detailed information regarding the management of patients undergoing vascular surgery. It is important to note that our center is a public hospital and faces certain limitations regarding equipment and resources, with a high volume of patients. As a result, we primarily rely on open surgery techniques for AAA repair. This reliance on open surgeries is not by choice but rather a necessity due to the limited availability of advanced endovascular equipment.

## 5 Conclusion

We observed an increase in the incidence of AAA surgeries in our center throughout the years, with the population growing towards younger population, while the incidence of rupture increasing towards older age groups. Based on the increase in the number of cases, further studies regarding detailed multicentral and longitudinal studies evaluation of the aneurysm operations based on etiology and outcome are warranted.

## Author Contributions

**Hamed Ghoddusi Johari:** conceptualization, supervision. **Keivan Ranjbar:** methodology, writing–review and editing. **Kimia Kassaee:** writing–original draft. **Seyed Mohammadali Hoseini:** writing–original draft, data curation. **Reza Shahriarirad:** conceptualization, methodology, data curation, writing–review and editing, formal analysis, project administration.

## Ethics Statement

The current investigation received approval from the medical ethics committee of Shiraz University of Medical Sciences (Ethical code: IR.SUMS.MED.REC.1402.039). Due to the retrospective nature of our study, the requirement for written informed consent was waived by the Ethics Committee of Shiraz University of Medical Sciences, and patient information was extracted from their hospital records. Authorization to conduct the study and access patient records was obtained from the administrators of Shiraz University of Medical Sciences, ensuring compliance with pertinent guidelines, regulations, the Declaration of Helsinki, and further approval by the university's ethics committee. All authors have read and approved the final version of the manuscript. The corresponding author, Reza Shahriarirad, had full access to all of the data in this study and takes complete responsibility for the integrity of the data and the accuracy of the data analysis. The lead author, Reza Shahriarirad, affirms that this manuscript is an honest, accurate, and transparent account of the study being reported; that no important aspects of the study have been omitted; and that any discrepancies from the study as planned (and, if relevant, registered) have been explained. Written informed consent for participation was waived due to the retrospective nature of our study by the Ethics Committee of Shiraz University of Medical Science.

## Consent

The authors have nothing to report.

## Conflicts of Interest

The authors declare no conflicts of interest.

## Transparency Statement

The lead author Reza Shahriarirad affirms that this manuscript is an honest, accurate, and transparent account of the study being reported; that no important aspects of the study have been omitted; and that any discrepancies from the study as planned (and, if relevant, registered) have been explained.

## Data Availability

The data that support the findings of this study are available on request from the corresponding author. The data are not publicly available due to privacy or ethical restrictions. The authors confirm that the data supporting the findings of this study are available within the article and its supplementary materials. The datasets used and analyzed during the current study are available from the corresponding author on reasonable request and with permission of the Research Ethics Committee of the School of Medicine‐Shiraz University of Medical Sciences.
